# Reduced TCA Flux in Diabetic Myotubes: Determined by Single Defects?

**DOI:** 10.1155/2012/716056

**Published:** 2012-03-18

**Authors:** Michael Gaster

**Affiliations:** ^1^Laboratory of Molecular Physiology, Department of Pathology, Odense University Hospital, 5000 Odense, Denmark; ^2^Department of Endocrinology, Odense University Hospital, 5000 Odense, Denmark

## Abstract

The diabetic phenotype is complex, requiring elucidation of key initiating defects. Diabetic myotubes express a primary reduced tricarboxylic acid (TCA) cycle flux but at present it is unclear in which part of the TCA cycle the defect is localised. In order to localise the defect we studied ATP production in isolated mitochondria from substrates entering the TCA cycle at various points. ATP production was measured by luminescence with or without concomitant ATP utilisation by hexokinase in mitochondria isolated from myotubes established from eight lean and eight type 2 diabetic subjects. The ATP production of investigated substrate combinations was significantly reduced in mitochondria isolated from type 2 diabetic subjects compared to lean. However, when ATP synthesis rates at different substrate combinations were normalized to the corresponding individual pyruvate-malate rate, there was no significant difference between groups. These results show that the primary reduced TCA cycle flux in diabetic myotubes is not explained by defects in specific part of the TCA cycle but rather results from a general downregulation of the TCA cycle.

## 1. Introduction

Type 2 diabetes (T2D) is a disorder characterised by impaired insulin secretion from beta cells and insulin resistance (IR) in peripheral target tissues. Skeletal muscle is a key tissue site of the IR. Although a number of abnormalities have been identified in skeletal muscle from T2D subjects, the exact molecular mechanisms for IR have not been established. The diabetic phenotype is complex, requiring elucidation of key initiating defects. We have previously described that myotubes established from T2D subjects conserve the diabetic phenotype [1]. Diabetic myotubes express an increased basal glucose oxidation, and incomplete lipid oxidation while complete lipid oxidation is reduced [2–6]. Recently we tested the hypothesis whether these alterations could be explained by a primarily reduced tricarboxylic acid (TCA) cycle flux and could show that this was reduced [7] and further that inhibition of TCA cycle flux in lean myotubes by malonate, a competitive inhibitor of the TCA cycle enzyme succinate dehydrogenase [8], is followed by a decline in acetate oxidation, complete palmitate oxidation and ATP level while glucose oxidation was unaffected, showing that an induced defect can force the lean phenotype in the direction of the diabetic phenotype [7]. A reduced TCA cycle flux has been shown in both insulin-resistant offspring of T2D patients [9] and exercising T2D patients *in vivo* [10]. Thus, a reduced TCA cycle flux may be responsible for part of the diabetic phenotype. The question arises in which part of the TCA cycle the defect is located, that is, whether the activity of single enzymes or the whole TCA cycle may be downregulated.

The intermediary metabolism of skeletal muscle *in vivo* is highly influenced by physical activity, ageing, hormonal status, and fiber type composition, rendering it difficult to determine the contribution of single factors to the alteration in mitochondrial metabolism. Cultured myotubes offer a unique model to distinguish between genetic and environmental factors in the etiology of insulin resistance and type 2 diabetes and to elucidate the molecular biological background [1–7, 11–17]. In the present study, we took advantage of our model of human myotubes to investigate whether substrates entering the TCA cycle at various points are oxidized differently in isolated mitochondria from myotubes established from lean and T2D subjects in order to clarify whether the activity of single enzymes or the entire TCA cycle may be downregulated.

## 2. Materials and Methods

### 2.1. Human Study Subjects 

Eight lean and eight obese patients with type 2 diabetes participated in the study. Their clinical characteristics have been published [11, 12] and are shown in Table 1. All subjects gave written, informed consent, and the local ethics committee of Funen and Vejle County approved the study.

### 2.2. Cell Culture

Cell cultures were established as previously described [18–20]. In brief, muscle tissue was minced, washed, and dissociated for 60 min by three treatments with 0.05% trypsin-EDTA. The cells obtained were seeded for upscaling on ECM-gel-coated dishes after 30 min of preplating. Growth medium contains DMEM supplemented with 2% FCS, 2% Ultroser G, 50 U/mL penicillin, 50 *μ*g/mL streptomycin, and 1.25 *μ*g/mL amphotericin B. Cells were subcultured twice before final seeding. At 75% confluence, the growth medium was replaced by basal medium (DMEM supplemented with 2% FCS, 50 U/mL penicillin, 50 *μ*g/mL streptomycin, 1.25 *μ*g/mL amphotericin B, and 25 pmol/L insulin) in order to induce differentiation. The cells were cultured in humidified 5% CO_2_ atmosphere at 37°C, and medium was changed every 2-3 days. Human myotubes established from each group were used for analysis at day eight after onset of differentiation.

### 2.3. Mitochondria Isolation and Preparation

Mitochondria were isolated from cultured human myotubes using the MACS mitochondria isolation kit from Miltenyi Biotech, Germany [13].

### 2.4. ATP

The ATP synthesis in isolated mitochondria was measured by luminescence at baseline and during ATP utilization by the hexokinase reaction that catalyses the production of glucose 6-phosphate from glucose and ATP. The mitochondrial buffers contained 300 mM sucrose, 10 mM KCl, 10 mM KH_2_PO_4_, 10 mM Tris-Base, 0.1 mM EDTA, and 0.035 mM ADP supplemented with different substrate combinations. Initially we investigated the following combinations: 1 mM pyruvate and 1 mM malate (PM buffer), 1 mM malate and 5 mM glutamate (MG buffer), 5 mM succinate and 10 *μ*M rotenone (SR buffer). In a second line of experiments we extended these with the following additional substrate combinations: 1 mM acetate 1 mM malate (AM buffer), 1 mM citrate 1 mM malate (CM buffer), or 1 mM/5 mM isocitrate 1 mM malate (IM buffer). All buffers were supplemented by 3 U/mL hexokinase and 1 mM glucose during conditions of ATP utilization. ATP production was measured after 0, 5, 10, 15, and 30 min, respectively. ATP was determined by luminescence (ATPlite, PerkinElmer, Turku, Finland) in 96-well plates and measured on a Microbeta counter (PerkinElmer, Finland) as previously described [21]. Mitochondrial ATP production was corrected for fading of luminescence of the luciferase used in the ATP assay and normalized per *μ*g of mitochondria protein and per minute.

### 2.5. Mitochondrial Mass

MitoTracker Green Probe (Molecular Probes, Eugene, OR) was used according to the manufacturer's instructions. Fluorescence was determined on a VICTOR plate reader model 1420-050 (PerkinElmer, Finland) with excitation and emission wavelength of 485 and 535, respectively, as described previously, [22]. Values were corrected for protein.

### 2.6. Statistical Analysis

Data in text, tables, and figures are given as mean ± SEM. Statistical analyses were performed with SPSS (version 17.0). ANOVA test was used to assess significant differences between groups. *P* ≤ 0.05 was considered to be significant.

## 3. Results

### 3.1. Mitochondrial ATP Synthesis

**Figure 1:**
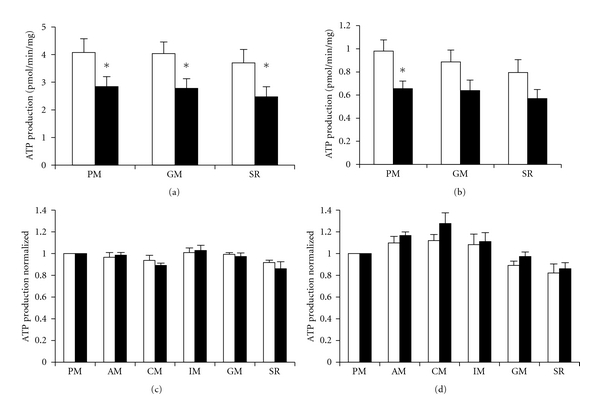
Rates of ATP synthesis in isolated mitochondria. The ATP synthesis rate was determined in isolated mitochondria of differentiated myotubes established from lean (open bars) and type 2 diabetic subjects (black bars). Isolated mitochondria were incubated with various substrates as indicated, supplemented with/without hexokinase as described in Section 2. (a) shows ATP synthesis in isolated mitochondria without hexokinase treatment. *P < 0.05 versus control. (b) shows ATP synthesis in isolated mitochondria with hexokinase treatment. *P < 0.05 versus control. (c) shows normalised ATP synthesis rates without hexokinase treatment. (d) shows normalised ATP synthesis rates with hexokinase treatment. Data are means ± SE, *n* = 8 in each group. acetate (A), citrate (C), isocitrate (I), glutamate (G), malate (M), pyruvate (P), rotenone (R), and succinate (S).

The ATP production in isolated mitochondria from diabetic myotubes was significantly reduced (*P* < 0.05) compared to mitochondria from lean in the presence of PM, MG, and SR (Figure 1(a)). When adding hexokinase to isolated mitochondria to simulate ATP demand, this was overall conserved (*P* < 0.01) and reached significance for PM (*P* < 0.05, Figure 1(b)).

### 3.2. Substrate Handling

The ATP synthesis in isolated mitochondria was measured at additional substrate combinations (acetate-malate (AM), citrate-malate (CM), isocitrate-malate (IM)). In order to show differences in substrate handling between mitochondria isolated from lean and obese T2D subjects ATP synthesis rates at different substrate combinations were normalized to values at PM. Mitochondria isolated from myotubes established from type 2 diabetic subjects did not reveal any differences in substrate handling for ATP synthesis compared to lean with or without the presence of hexokinase (*P* > 0.19) (Figures 1(c) and 1(d)).

### 3.3. Mitochondrial Mass

**Figure 2:**
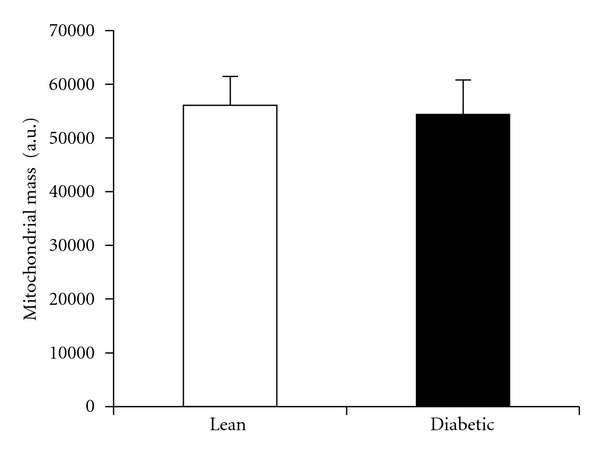
Mitochondrial mass. Mitochondrial mass was determined by MitoTracker Green fluorescence in myotubes established from lean (open bar) and T2D subjects (black bar). Data are means ± SE, *n* = 8 in each group.

The mitochondrial mass in myotubes established from lean and obese diabetic subjects was measured in order to identify group differences in mitochondrial content. We could not detect any significant differences in mitochondrial mass between groups (*P* = 0.86, Figure 2). 

## 4. Discussion

Pyruvate, acetate, malate, citrate, isocitrate, glutamate, and succinate enter the TCA cycle at different sites before they are oxidised, creating a transmembrane potential, powering the phosphorylation of ADP to ATP. Their energy is predominantly transferred to complex I of the electron transport chain (ECT) for all substrates except for the combination succinate-rotenone which preliminary deliver to complex II. As in our previous studies of ATP production in isolated mitochondria from diabetic myotubes, the ATP production was reduced compared to mitochondria from lean [13, 14]. To search for TCA cycle defects in diabetic mitochondria, we exposed isolated mitochondria for substrates with different TCA cycle entries to locate TCA cycle defects. All entrance points to the TCA cycle gave similar normalised ATP synthesis rates in the two groups indicating that there may not be a major single primary defect present in the TCA cycle and the reduced TCA cycle flux in diabetic mitochondria seems to be based on a general TCA cycle downregulation. Both complex I and II substrates gave the same results indicating that the defect is either downstream from complex II, that is, at the sites of cytochrome C oxidase or ATP synthase, or a general reduction in TCA cycle flux. Recent evidence from global approaches such as microarray gene expression analysis has suggested that the basis for reduced mitochondrial ATP production in skeletal muscle from T2D could be a functional impairment in oxidative phosphorylation (OXPHOS) [23, 24]. However, transcriptional profiling of myotubes established from T2D subjects did not show evidence of a primary defect in OXPHOS genes [22]. Moreover, we have previously determined the uncoupled respiration rate in myotubes established from lean, obese, and T2D subjects and found no significant differences between groups, suggesting that the ETC did not express intrinsic defects [12]. Contradicting a primary major defect in the oxidative phosphorylation in human myotubes, the basal glucose oxidation decreased when oxidative phosphorylation was either inhibited by Antimycin A (Complex III inhibitor) or oligomycin (F0-F1 inhibitor) in myotubes established from lean, obese, and T2D subjects [6] showing that lean, obese, and diabetic react in the same manner. However, diabetic myotubes express an increased basal glucose oxidation during normophysiological condition and still expressed an increased basal glucose oxidation compared to lean myotubes, despite inhibition of the oxidative phosphorylation, indicating that defects in the oxidative phosphorylation were not the major mechanism to explain primary changes in diabetic myotube metabolism [6]. To identify and quantify changes in protein abundance between human myotubes obtained from lean, obese, and type 2 diabetic subjects we did a quantitative proteomic study [15]. Despite a clear diabetic phenotype in diabetic myotubes, only twelve proteins were differentially expressed between the three different groups. Proteins from all major pathways known to be important in T2D were well characterized including the TCA cycle, lipid oxidation, oxidative phosphorylation, the glycolytic pathway, and glycogen metabolism. None of these enzymes were found to be regulated at the level of protein expression or degradation, suggesting that above differences in ATP synthesis may not be found on the protein expression level. Taken together, the above data indicates that there are no intrinsic single defects in the TCA cycle which could explain the reduced TCA cycle flux in diabetic mitochondria.

A general downregulation of the TCA cycle can be obtained at the level of posttranslational modification (PTM) of TCA cycle proteins or through changes in intramyocellular energy-redox state controlling the overall TCA cycle flux rate. Less is known about mitochondrial phosphatases and kinases. Studies indicate that cytosolic kinases may be translocated into the mitochondria that is, PKA, PKC, or AKT. A recent paper reports that the activity of mitochondrial aconitase (a TCA cycle enzyme) in type 1 diabetic rat hearts is regulated by PKC*β*_2_, whose activity is dependent on the degree of phosphorylation [25]. Augmented phosphorylation of mitochondrial aconitase was associated with a reduced TCA cycle flux, based on an increased reverse activity, while the forward reaction was normal. That PTM could be part of the explanation for a reduced TCA cycle flux in diabetic mitochondria is supported by our previous finding of a 14% reduced citrate synthase activity in diabetic myotubes [12] which cannot be explained by changes in protein expression [15]. Additional phosphoproteomic studies are required to test whether the impaired TCA cycle flux, in diabetic mitochondria, is based on posttranscriptional modifications of TCA cycle enzymes.

A more overall regulation of the TCA cycle in human myotubes may be based on the mitochondrial matrix phosphorylation potential (Pi + ADP/ATP) and the pyridine nucleotide redox poise and concentration (NADH/NAD+). Increasing substrate availability is followed by increasing concentrations of reduction equivalents and ATP which, through allosteric inhibition, can downregulate the TCA cycle flux. Previously we determined the energy charge, the level of ATP, ADP, and AMP in myotubes established from lean, obese, and obese type 2 diabetic subjects at normophysiological conditions and could not verify differences between groups [26] indicating that the energy state may not account for differences in the TCA cycle flux between groups.

Increased oxidative stress has been implicated in the development of insulin resistance in type 2 diabetes by both indirect and direct evidence based on increased damage of DNA, lipids, and proteins [27]. Recently we measured hydrogen peroxide production/mitochondrial mass and found it significantly reduced in diabetic myotubes compared to lean controls indicating that an increased ROS production may not be the main mechanism accounting for a reduced TCA flux in diabetic mitochondria [16].

The reduced but similar ATP production on different substrates in diabetic mitochondria could point to reduced substrate availability as explanation for obtained differences, that is, based on impaired transport/interchange of substrates across the inner mitochondrial membrane. Several substrates were used in the present study requiring different transporters indicating that transport/interchange of substrate and intermediates could be generally impaired in diabetic mitochondria. However, we have recently described that the inner mitochondrial membrane potential was conserved in diabetic mitochondria [16]. Further studies are required to clarify whether substrate availability may be part of the responsible mechanism for a reduced ATP production in diabetic mitochondria.

An overall reduced TCA flux in diabetic myotubes compared to lean myotubes could be based on a reduced mitochondrial mass in diabetic myotubes. We addressed the question by measuring the mitochondrial mass in myotubes established from lean and type 2 diabetic subjects and could not show significant differences between groups.

In summary, we tested the hypothesis that the reduced TCA cycle flux in diabetic mitochondria was based on site-specific TCA cycle defects but we could not find evidence for this. The primary reduced TCA cycle flux in diabetic myotubes is explained by a general downregulation of the TCA cycle. We hypothesize that the impaired TCA cycle flux, in diabetic mitochondria, is based on posttranscriptional modifications of TCA cycle enzymes.

## Figures and Tables

**Table 1:** *In vivo* characteristics.

	Control	Diabetic
*n*	8	8
Age (years)	51 ± 1	49 ± 2
Weight (kg)	69.7 ± 3.4	101.8 ± 5.1*
BMI (kg/m^2^)	24.4 ± 0.6	34.0 ± 1.4*
Fasting plasma glucose (mM)	5.7 ± 0.1	10.1 ± 0.8*
Fasting serum insulin (pM)	26.5 ± 6.9	94.6 ± 10.1*
Glucose infusion rate (mg/min)	388.8 ± 22.2	100.0 ± 11.9*
HbA_1c_ (%)	5.5 ± 0.1	7.7 ± 0.5*

Data are means ± SE. *Significantly different from the controls (*P* < 0.05).
